# **Motor-Related Neural Dynamics are Modulated by Regular Cannabis Use Among People with HIV**

**DOI:** 10.1007/s11481-025-10219-0

**Published:** 2025-06-06

**Authors:** Lauren K. Webert, Mikki Schantell, Lucy K. Horne, Jason A. John, Ryan Glesinger, Jennifer O’Neill, Maureen Kubat, Anna T. Coutant, Grace C. Ende, Sara H. Bares, Pamela E. May-Weeks, Tony W. Wilson

**Affiliations:** 1 Institute for Human Neuroscience, Boys Town National Research Hospital, 14090 Mother Teresa Ln., Boys Town, NE 68010 USA; 2 College of Medicine, University of Nebraska Medical Center (UNMC), Omaha, NE USA; 3 Divison of Infectious Diseases, Department of Internal Medicine, UNMC, Omaha, NE USA; 4 Department of Neurological Sciences, UNMC, Omaha, NE USA; 5 Department of Pharmacology & Neuroscience, Creighton University, Omaha, NE USA

**Keywords:** Cognitive interference, Motor cortex, Gamma, Beta, Oscillations, Spontaneous activity, Magnetoencephalography, MEG

## Abstract

Recent work has shown that people with HIV (PWH) exhibit deficits in cognitive control and altered brain responses in the underlying cortical networks, and that regular cannabis use has a normalizing effect on these neural responses. However, the impact of regular cannabis use on the neural oscillatory dynamics underlying motor control deficits in PWH remains less understood. Herein, 102 control cannabis users, control nonusers, PWH who regularly use cannabis, and PWH who do not use cannabis performed a motor control task with and without interference during high-density magnetoencephalography. The resulting neural dynamics were examined using whole-brain, voxel-wise statistical analyses that examined the impact of HIV status, cannabis use, and their interaction on the neural oscillations serving motor control, spontaneous activity during the baseline period, and neurobehavioral relationships. Our key findings revealed cannabis-by-HIV group interactions in oscillatory gamma within the prefrontal cortices, higher-order motor areas, and other regions, with the non-using PWH typically exhibiting the strongest gamma interference responses. Cannabis-by-HIV interactions were also found for oscillatory beta in the dorsal premotor cortex. Spontaneous gamma during the baseline was elevated in PWH and suppressed in cannabis users in all regions exhibiting interaction effects and the left primary motor cortex, with spontaneous levels being correlated with behavioral performance. These findings suggest that regular cannabis use has a normalizing effect on the neural oscillations serving motor control and the abnormally elevated spontaneous gamma activity that has been widely replicated in PWH, which may suggest that cannabis has at least some therapeutic utility in PWH.

**Supplementary Information:**

The online version contains supplementary material available at 10.1007/s11481-025-10219-0.

## Graphical Abstract

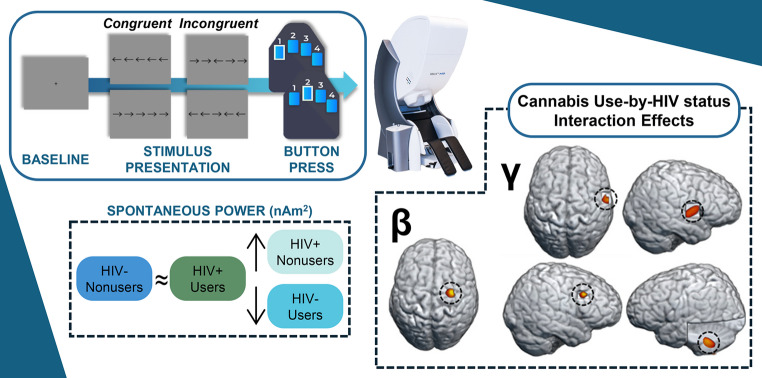

## Introduction

Despite remarkable advances in HIV treatment, people with HIV (PWH) still face a greater risk of developing cognitive impairment relative to the general population (Antinori et al. 2007; Heaton et al. 2011; Winston and Spudich 2020). Previous studies have linked these neurocognitive impairments to aberrant neuroinflammatory activity in PWH (Purohit et al. 2014; Spooner et al. 2021c), although other processes also contribute to cognitive decline, and the precise origins remain incompletely understood (Wilson et al. 2019; Ellis et al. 2023). Of note, a disproportionate number of PWH use cannabis, which has been shown to alleviate some of these inflammation-related symptoms (Manuzak et al. 2018; Watson et al. 2021). Cannabis is an agonist to cannabinoid receptors (CB1 and CB2) on immune cells which can modulate the release of inflammatory cytokines (Purohit et al. 2014; Ellis et al. 2020; Chou et al. 2022), and multiple studies have linked cannabis to reduced neuro-inflammation in PWH (Manuzak et al. 2018; Ellis et al. 2020). Along these lines, several recent studies of PWH have linked regular cannabis use to the normalization of spontaneous and oscillatory activity (Christopher-Hayes et al. 2021; Schantell et al. 2022a), which a large number of studies have shown to be aberrant in PWH, especially in those with cognitive impairment (Arif et al. 2020; Becker et al. 2013; Casagrande et al. 2021; Groff et al. 2020; Lew et al. 2018; Schantell, Taylor, et al., 2022b; Spooner et al. 2018; Spooner, Wiesman et al. 2020; Wiesman et al. 2018).

Past neuroimaging studies of HIV have tended to focus on resting-state activity (Thomas et al. 2013; Becker et al. 2013) or the neural responses serving cognitive task performance (Wilson et al. 2016, 2017; Wiesman et al. 2018; Lew et al. 2018; Schantell et al. 2022b), with far fewer studies examining motor control (Wilson et al. 2013). In healthy populations, neurophysiological studies of motor control have identified a series of specific neural oscillatory responses required for the planning, execution, and termination of movement. The earliest of these oscillatory neural responses is a strong beta band (14–30 Hz) response termed the peri-movement beta event-related desynchronization (ERD), which starts prior to and extends through the duration of the movement (Heinrichs-Graham and Wilson 2015; Wilson et al. 2016). Next, there is a strong event-related synchronization (ERS) in the gamma band (60–90 Hz) that coincides with movement onset (Cheyne et al. 2008; Spooner and Wilson 2022, 2023). Following movement termination, a second beta band response termed the post-movement beta rebound (PBMR) is observed (Heinrichs-Graham et al. 2017).

As noted above, neuroimaging studies examining motor control in PWH are exceedingly rare, with only a few studies focusing on virally-suppressed patients and three of these using magnetoencephalography (MEG; Spooner et al. 2023, 2024; Wilson et al. 2013). Utilizing a simple finger-tapping task, Wilson et al. (2013) found that PWH exhibited significantly reduced peri-movement beta ERD responses in the primary motor cortex and supplementary motor area (SMA) compared to healthy controls. More recently, such aberrant beta ERD responses in the primary motor cortices of PWH have been linked to HIV-related dysfunction in the mitochondrial redox environment (Spooner et al. 2023, 2024). These studies used a more complicated motor sequencing task and found that PWH performed more poorly and exhibited an altered relationship among mitochondrial function, reactive oxygen species, and neural oscillations compared to seronegative controls (Spooner et al. 2021b, 2023). Beyond studies focusing on motor control, resting-state MEG work has shown altered spontaneous beta activity in the motor cortices of virally suppressed PWH compared to controls (Becker et al. 2013), and multiple structural MRI studies have found reduced gray matter thickness in areas associated with motor control in PWH compared to healthy controls (Zhou et al. 2017; Lew et al. 2021).

While the available evidence does support altered motor-related beta activity in PWH, to our knowledge, no study to date has examined high-frequency gamma oscillations in PWH, which are thought to support motor execution and be critical for higher-order cognitive functions more generally (Heinrichs-Graham et al. 2018; Spooner et al. 2019; Wiesman et al. 2021a; Arif et al. 2021). This is surprising, as previous neuroimaging studies have revealed that PWH have significantly elevated spontaneous gamma-band activity compared to healthy controls in somatosensory and visual occipital cortices, which in some cases has been shown to distinguish cognitively-impaired and unimpaired PWH (Spooner et al. 2018, 2020; Wiesman et al. 2018; Lew et al. 2018; Casagrande et al. 2022). Such aberrations are especially notable in this context, as several MEG studies have shown that cannabis use is associated with sharply suppressed spontaneous gamma activity (Arif et al. 2021; Christopher-Hayes et al. 2021; Webert et al. 2024).

In this study, we investigated the impact of cannabis and HIV on the oscillatory dynamics serving motor control in a sample of regular cannabis users and nonusers with and without HIV (i.e., four groups). To this end, we used MEG imaging and advanced oscillatory analyses to quantify beta and gamma responses serving motor control in the context of a cognitive interference task. We used a cognitive interference motor task because prior work has shown that PWH are especially sensitive to such interference (Lew et al. 2018; Schantell et al. 2022a; Meehan et al. 2023) and normative work has shown that oscillatory motor responses are generally robust during such tasks (Heinrichs-Graham et al. 2018; Spooner et al. 2021a; Arif et al. 2024). Based on previous literature (Spooner et al. 2018, 2020; Wiesman et al. 2018; Christopher-Hayes et al. 2021; Schantell et al. 2022a; Springer et al. 2023), we hypothesized that PWH who do not use cannabis would exhibit altered oscillatory neural responses during task performance and abnormally elevated spontaneous activity during the baseline period in the same brain regions, while PWH who regularly use cannabis would have relatively normal oscillatory motor responses and spontaneous neural activity in these brain regions.

## Methods

### Participants

A total of 108 participants (33 HIV- nonusers, 36 HIV- cannabis users, 20 HIV + nonusers, and 19 HIV + cannabis users) were selected from a larger ongoing study based on them having successfully completed a structured substance use interview, a neuropsychological assessment, a structural MRI, and the flanker task during MEG, and not meeting the exclusion criteria described below. Cannabis users and nonusers with HIV were recruited from the University of Nebraska Medical Center’s HIV Clinic, and control users and nonusers were recruited from the Omaha metropolitan area. PWH were required to be on an effective antiretroviral therapy (ART) regimen defined as an HIV RNA viral load of less than 50 copies/mL within three months of participation in the study. At the time of neuropsychological testing, all controls were confirmed seronegative using the OraQuick *ADVANCE*^®^ Rapid HIV-1/2 Antibody Test. As part of the inclusion criteria for this study, cannabis users were required to have used cannabis at least two times per week for the past six months and reported less than monthly use of other recreational substances. Nonusers were excluded if they used any substances other than alcohol in the past 12 months or had a history of using any substance other than experimental use (i.e., once or twice). Exclusion criteria included any neurological or psychiatric disorder, history of head trauma, current pregnancy, history of substance use disorder in nonusers, or ferrous metallic implants that could interfere with MEG data acquisition. The Institutional Review Board reviewed and approved this protocol. All participants gave written informed consent following a detailed description of the study.

### Neuropsychological Assessment

To more fully phenotype the sample, all participants underwent a neuropsychological assessment designed to assess for HIV-associated cognitive impairment in accordance with the Frascati criteria (Antinori et al. 2007). The test battery assessed the following cognitive domains: *learning*,* memory*,* executive functioning*,* processing speed*,* attention*, and *motor dexterity* (Heaton et al. 2004). Cognitive impairment was assigned per the Frascati criteria (Antinori et al. 2007) by a neuropsychologist using the demographically corrected composite domain z-scores along with a modified version of the Lawton and Brody (Lawton and Brody 1969) Instrumental Activities of Daily Living scale to assess perceived functional impairment.

### Substance Use Assessments

All cannabis users completed substance use assessments including a thorough interview regarding their lifetime and current (within the last 12 months) substance use history using the NIDA Quick Screen (Version 1), NIDA-Modified Alcohol, Smoking, and Substance Involvement Screening Test (NM-ASSIST; Version 2), and Module E of the Structured Clinical Interview for the Diagnostic and Statistical Manual, 5th Edition (SCID-5). Participants also completed self-report questionnaires, including the Cannabis Use Disorders Identification Test – Revised (CUDIT-R), the Alcohol Use Disorders Identification Test - Concise (AUDIT-C), and a custom cannabis use questionnaire detailing their cannabis use methods and history. Participants also provided a urine sample to confirm no recent use of substances other than cannabis. Nonusers were interviewed on past and current substance use using a standardized medical history interview, and alcohol use was assessed using the AUDIT-C.

### MEG Experimental Paradigm

**Fig. 1:**
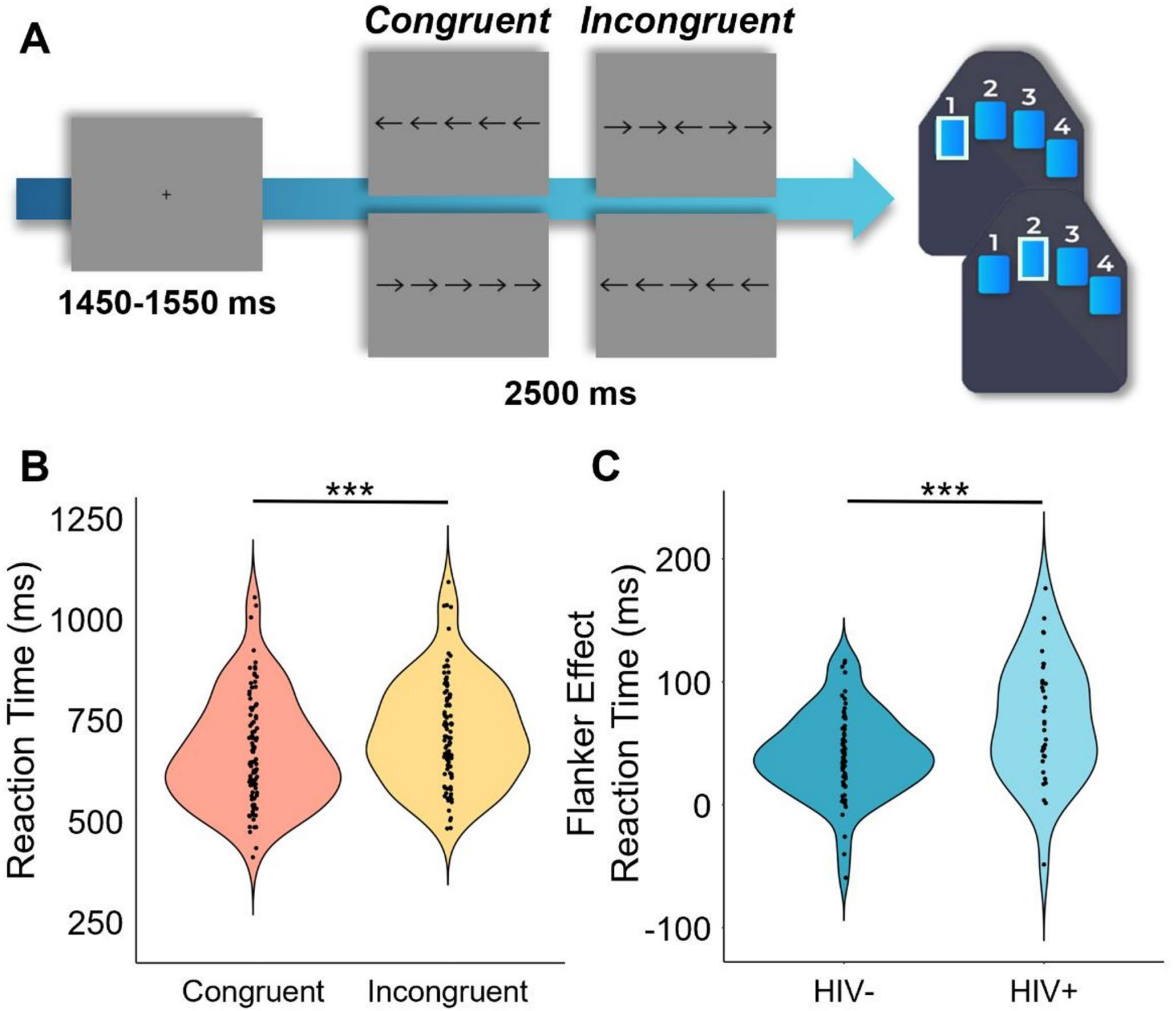
Experimental Paradigm and Behavioral Results. (**A**) An illustration of the flanker arrow paradigm. Each trial had a fixation period lasting on average 1500 ms (variable ISI: 1450–1550 ms) and a stimulus-presentation period lasting 2500 ms, which consisted of one of the four shown options. (**B**) Reaction time (in ms) across all participants is shown on the *y*-axis with condition on the *x*-axis. Across all groups, participants responded slower during incongruent relative to congruent trials (i.e., classic flanker effect; *p* = 2.22 × 10^− 21^). (**C**) Neither the main effects of cannabis nor HIV group were significant, but the condition-by-HIV group interaction was, and follow-up testing showed that PWH exhibited a larger flanker interference effect than seronegative participants (*p* =.007). ***p ≤.001

Participants were seated in a nonmagnetic chair within a magnetically shielded room and completed a 14-minute arrow-based flanker task with 200 pseudorandomized trials. A fixation cross was centrally presented for 1450 to 1550 ms, which was followed by the presentation of a row of five arrows for 2500 ms. Participants responded using their right hand whether the middle arrow was pointing to the left (index finger) or right (middle finger; Fig. 1A). The trials were equally divided between congruent and incongruent conditions, with left and right arrows being equally represented in each of the conditions. The number of trials used in this study was based on previous motor control studies using the same paradigm (Heinrichs-Graham et al. 2018; Spooner et al. 2021a; Arif et al. 2024; Son et al. 2024). Reaction time and accuracy measures were collected and used for behavioral analysis.

### MEG and MRI Data Acquisition

Functional MEG data were collected using a MEGIN MEG system (Helsinki, Finland) equipped with 306 sensors (204 planar gradiometers, 102 magnetometers) using a 1 kHz sampling rate and an acquisition bandwidth of 0.1–330 Hz in a one-layer magnetically shielded room with active shielding engaged. Prior to MEG acquisition, four coils were attached to the participant’s head and localized along with fiducial and scalp surface points using a three-dimensional (3D) digitizer (FASTRAK, Polhemus Navigator Sciences, Colchester, Vermont). Once the participants were positioned for MEG recording, an electric current with a unique frequency label (e.g., 322 Hz) was fed to each of the four coils, thus inducing a measurable magnetic field and thereby allowing each coil to be localized in reference to the MEG sensor array throughout the recording session. Structural T1-weighted MRI data were collected using a 3D-fast-field echo sequence on a Philips Achieva 3.0T X-Series scanner with an eight-channel head coil. The parameters for the 3D-fast-field echo sequence were as follows: TR: 8.09 ms; TE: 3.7 ms; field of view: 24 cm; matrix: 256 × 256; slice thickness: 1 mm with no gap; in-plane resolution: 0.9375 × 0.9375 mm; sense factor: 1.5 (Lew et al. 2021).

### MEG and MRI Processing

MEG and MRI data processing closely followed previously reported pipelines (Wiesman and Wilson 2020a; Wiesman et al. 2021b; Webert et al. 2024). The structural MRI data were aligned parallel to the anterior and posterior commissures and transformed into standardized Talairach coordinate space. MEG data were subjected to environmental noise reduction and corrected for head motion using the signal space separation method with a temporal extension. Only data from the 204 planar gradiometers were used for further analysis. All MEG and MRI data were further processed in BESA (Research: Version 7.1; MRI: Version 3.0; Statistics: Version 2.1). Cardiac and ocular artifacts were regressed out of the MEG data using signal space projection.

### MEG Time-Frequency Transformation & Sensor Space

The resulting artifact-corrected data were then bandpass filtered from 0.5 to 150 Hz, notch filtered at 60 Hz, and divided into 4000 ms epochs (-2000 to 2000 ms), with 0.0 s defined as movement onset and the baseline period being − 1800 to -1000 ms. Epochs containing artifacts were rejected based on a fixed threshold method that was set per participant and supplemented with visual inspection. Briefly, in MEG, the raw signal amplitude is strongly affected by the distance between the brain and the MEG sensor array, as the magnetic field strength falls off sharply as the distance from the current source (i.e., brain) increases. To account for this source of variance across participants, as well as other sources of variance, we used an individualized threshold based on the signal distribution for both amplitude and gradient to reject artifacts. There were no differences in the number of accepted trials by group (control nonuser = 179.82 trials, control cannabis user = 179.21 trials, non-using PWH = 179.88 trials, cannabis using PWH = 181.00 trials, *F* = 0.06, *p* =.982) or by condition (congruent = 89.90 trials, incongruent = 89.93 trials, *t* = 0.07, *p* =.943). The average amplitude threshold across all participants was 1178.68 (SD = 395.78) fT/cm, and the average gradient threshold was 246.23 (SD = 124.91) fT/(cm*ms). Artifact-free epochs were transformed into the time-frequency domain using complex demodulation (Kovach and Gander 2016), and the resulting spectral power estimations per sensor were averaged across trials to generate time-frequency plots of mean spectral density. These sensor-level data were then normalized with respect to the mean baseline power (i.e., -1800 to -1000 ms). Time-frequency windows for subsequent source imaging were identified across all participants and conditions using a data-driven approach. Specifically, paired-sample t-tests against baseline were computed for each time-frequency bin across the entire frequency (4–100 Hz) and time range (-1800 to + 2000 ms) for each MEG sensor. The dimensions of these bins were determined by our frequency resolution of 1 Hz and time resolution of 50 ms. The resulting spectrograms of *t*-values were thresholded at an a-priori defined alpha level (*p* <.001) to identify clusters of significant bins (i.e., spectrally, spatially, and/or temporally neighboring), and cluster *t*-values were then derived by summing the values of each bin per cluster. Nonparametric permutation testing was then used to derive a distribution of cluster-values, and the significance level of the observed cluster(s) were tested directly using this permuted distribution, which was the result of 10,000 permutations. Based on this cluster-based permutation analysis, only the time-frequency windows that contained significant oscillatory deviations from baseline at the *p* <.001, corrected, threshold across all participants and conditions were subjected to source imaging (i.e., beamforming; Ernst 2004; Maris and Oostenveld 2007; Proskovec et al. 2018; Wiesman et al. 2018; Wiesman and Wilson 2020a).

### MEG Source Imaging

Each participant’s MEG data were co-registered with their structural T1-weighted MRI prior to source imaging analysis. We then used the dynamic imaging of coherent sources (DICS) beamformer to image oscillatory activity in the time-frequency windows of interest in each condition per participant (Gross et al. 2001). Note that these time-frequency windows of interest were based on the sensor-level statistical analysis described in the previous section. Specifically, we used task active and baseline periods of equal duration and bandwidth to compute noise-normalized source power per voxel, with the resulting pseudo-*t* maps reflecting power differences (i.e., active versus baseline) per voxel (resolution: 4 × 4 × 4 mm). These maps were then transformed into standardized space by applying the same transform that was applied to the native space structural images per participant and spatially resampled. To identify the origin of the sensor-level neural oscillations, grand-average maps across all participants and both conditions were computed per oscillatory response.

### Peak Voxel time Series

Peak voxel time series were extracted from the grand-averaged oscillatory maps and the whole-brain statistical maps probing the cannabis-by-HIV interaction (see below) to assess for group differences in spontaneous activity during the baseline period. Virtual sensor data were computed by applying the sensor-weighting matrix derived through the forward computation to the preprocessed signal vector, which yielded two orthogonal time series corresponding to the location of interest (i.e., peak voxels exhibiting significant interactions and peak voxels from the grand-averaged oscillatory maps). Next, these virtual sensor data were decomposed into time-frequency space to derive a single temporal envelope of the signal corresponding to the frequency window identified through the MEG sensor-level statistical analyses. This resulted in an absolute power time series for each peak voxel per participant, which was used to test for differences in spontaneous (baseline) activity.

### Statistical Analyses

For behavioral analyses (i.e., reaction time and accuracy), 2 × 2 × 2 ANOVAs with a within-subject factor of condition (i.e., congruent and incongruent) and between-subject factors of cannabis use (i.e., users and nonusers) and HIV status (i.e., PWH and controls) were used to identify main effects and interactions. Post-hoc *t*-tests were used to follow-up significant interaction effects. For the voxel-level analyses, we computed whole-brain flanker interference maps per participant by subtracting the congruent from the incongruent map (i.e., incongruent map – congruent map) and then conducted 2 × 2 whole-brain ANOVAs (cannabis use-by-HIV status) per oscillatory response to probe main effects and interactions involving neural oscillatory activity. The resulting whole-brain statistical maps were thresholded at *p* <.005 and we accounted for multiple comparisons using a cluster threshold (*k*) of 5 contiguous voxels (i.e., > 300 mm^3^), based on Gaussian random fields theory (Poline et al. 1995; Worsley et al. 1996, 1999). Group differences in pre-stimulus spontaneous activity were also assessed using a 2 × 2 ANOVA (cannabis use-by-HIV status) per region. Neurobehavioral and substance use correlations were assessed using Pearson correlations. All whole-brain statistical analyses were computed using the Statistical Parametric Mapping (SPM12) software and other statistical analyses were conducted in IBM SPSS v.25. To reduce the impact of extreme values, participants with values 3 SDs above or below their respective group means were excluded for each analysis.

### Data Availability Policy

De-identified data have been made available to the public through the Collaborative Informatics and Neuroimaging Suite (COINS) database.

## Results

### Participant Characteristics

Of the 108 participants included in this study, 102 participants were included in the final analyses following exclusions for poor performance or excessive MEG artifacts (3 non-using PWH, 1 cannabis using PWH, and 2 control users). The four groups had comparable demographic characteristics (Table 1), although there was a trending group difference in age. Given this, we re-computed all primary analyses using age as a covariate of no interest. HIV-related measures such as years since HIV diagnosis, years on ART, nadir CD4 counts, and current CD4 counts were similar between the two groups of PWH. All PWH were virally suppressed (HIV viral load < 50 copies/mL) as part of the inclusion criteria.

**Table 1:** Participant demographics and clinical indices

	HIV- Nonuser *n* = 33	HIV- User *n* = 34	HIV + Nonuser *n* = 17	HIV + User *n* = 18	*p*-value
Sex (Male/Female)	16/17	20/14	8/9	13/5	0.34
Age (years)	36.73 (11.22)	34.49 (10.44)	40.33 (11.69)	42.74 (12.21)	0.06
AUDIT-C Score	2.52 (1.87)	3.64 (2.32)	2.29 (1.99)	2.72 (2.08)	0.09
CUDIT Score	-	13.24 (4.61)	-	12.89 (4.58)	0.80
CD4 Nadir (cells/µL)	-	-	290.29 (169.75)	309.00 (191.95)	0.78
Current CD4 (cells/µL)	-	-	820.00 (247.81)	783.72 (351.45)	0.73
Years Since HIV Diagnosis	-	-	7.24 (3.62)	8.85 (6.14)	0.38
Years on ART	-	-	5.88 (3.43)	6.68 (6.81)	0.68

Note. Means and standard deviations are displayed for all variables except sex, which is displayed as the number of males and females, respectively. AUDIT-C – Alcohol Use Disorders Identification Test – Concise; CUDIT-R – Cannabis Use Disorders Identification Test – Revised; cART – Antiretroviral Therapy

### Flanker Task Performance

Overall, participants performed well on the modified flanker task, with high accuracy (HIV- Nonuser: *M* = 97.92%, *SD* = 0.04; HIV- User: *M* = 96.79%, *SD* = 0.05; HIV + Nonuser: *M* = 99.26%, *SD* = 0.01; HIV + User: *M* = 97.58%, *SD* = 0.04) and fast reaction times (HIV- Nonuser: *M* = 700.48 ms, *SD* = 152.87 ms; HIV- User: *M* = 664.08 ms, *SD* = 122.49 ms; HIV + Nonuser: *M* = 710.18 ms, *SD* = 103.14 ms; HIV + User: *M* = 719.00 ms, *SD* = 120.96 ms) across conditions. To assess the relationship between the flanker task conditions, cannabis use, HIV status, and their interaction on behavioral performance, we conducted two 2 × 2 × 2 ANOVAs. We found no significant main effects of condition (*F* = 0.79, *p* =.377), cannabis use (*F* = 2.45, *p* =.122), HIV status (*F* = 1.40, *p* =.240), nor their interaction effects on accuracy (condition-by-cannabis: *F* = 0.01, *p* =.377; condition-by-HIV: *F* = 2.26, *p* =.136; cannabis-by-HIV: *F* = 0.09, *p* =.760; condition-by-cannabis-by-HIV: *F* = 0.63, *p* =.430). The 2 × 2 × 2 ANOVA on reaction time revealed a significant main effect of condition (*F* = 172.29, *p* = 2.58 × 10^− 23^), indicating that across all groups, participants responded more slowly on incongruent relative to congruent trials (i.e., the classic flanker interference effect; *t* = -12.12, *p* = 2.22 × 10^− 21^; Fig. 1). Both group main effects were not significant (cannabis effect: *F* = 0.26, *p* =.613; HIV effect: *F* = 1.42, *p* =.237), but the condition-by-HIV group interaction was significant (*F* = 11.24, *p* =.001; Fig. 1). Follow-up testing of this two-way interaction indicated that PWH did not differ from seronegative controls in reaction time during congruent (*t* = − 0.67 *p* =.133) or incongruent (*t* = -1.73, *p* =.146) trials, but that the flanker interference effect (i.e., difference in reaction times between conditions) was larger in PWH (*t* = -3.34, *p* =.007; Fig. 1) relative to seronegative controls. No other interaction effects on reaction time were significant (condition-by-cannabis: *F* = 1.87, *p* =.175; cannabis-by-HIV: *F* = 0.69, *p* =.407; condition-by-cannabis-by-HIV: *F* = 0.13, *p* =.723). Of note, re-computing these ANOVAs with age as a covariate of no interest in an ANCOVA framework did not change the key findings.

### Sensor-Level Neural Oscillatory Responses

**Fig. 2:**
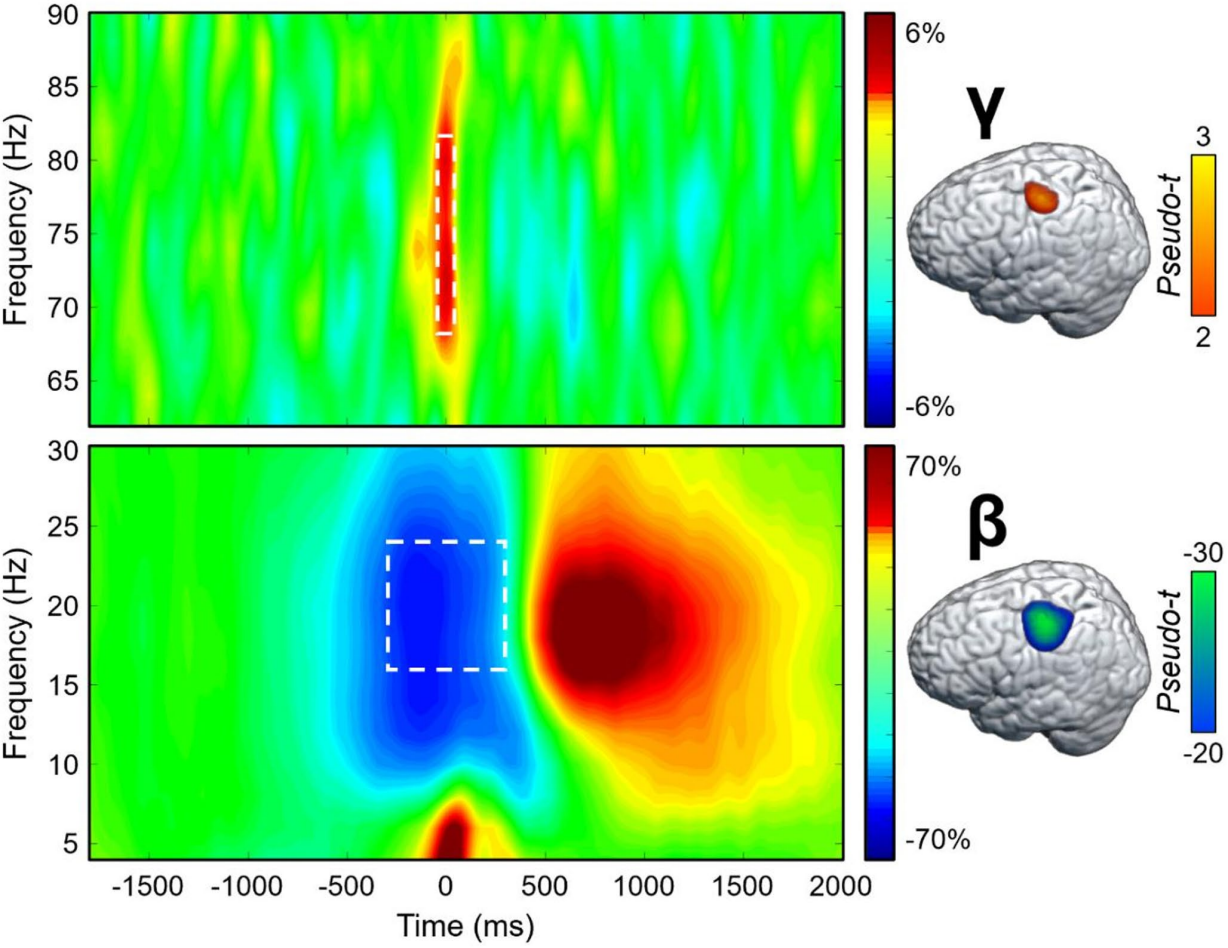
Motor-related neural oscillatory responses. (**Left**): Grand-averaged time-frequency spectrograms from MEG sensors near the precentral gyrus (top: MEG0432; bottom: MEG0443) exhibiting a significant motor-related gamma response (top; 68–82 Hz, -75 to 75 ms) and a peri-movement beta ERD response (bottom; 16–24 Hz, -300 to 300 ms). The spectrograms display frequency (Hz) on the *y*-axis and time (ms) on the *x*-axis. Signal power is expressed as a percent difference from the baseline period, with the color legend shown to the right of the spectrogram. Note that the post-movement beta rebound (PMBR) response can also be seen, but this was not further examined since it happened after the movement, and we were interested in the motor planning and execution aspects of the task. (**Right**): Grand-averaged beamformer images (pseudo-*t*) across all participants for each significant time-frequency component

To identify the time-frequency windows for beamformer imaging of the MEG signals, paired t-tests against baseline followed up with nonparametric permutation testing for multiple comparison correction were conducted on the sensor-level spectrograms collapsed across both conditions and all participants. These analyses revealed a significant increase in gamma power (68–82 Hz) relative to the baseline period between − 75 and 75 ms, and a significant peri-movement beta ERD (16–24 Hz) from − 300 to 300 ms (both *p*s < 0.001, corrected; Fig. 2). These windows were imaged per condition and participant. The resulting maps were first grand-averaged across all participants and both conditions to identify the primary origins of each oscillatory response, which revealed clusters in the left primary motor cortex for both significant time-frequency components (Fig. 2). Next, the whole-brain beta and gamma maps per condition were used in the statistical analyses described below to identify the impact of cannabis and HIV on neural oscillatory activity. Note that the post-movement beta rebound (PMBR) was also robust in sensors near the sensorimotor strip, but this was not examined because it began after movement termination and the current study was focused on how the cognitive components of the task affect motor planning and execution.

### Whole-Brain Oscillatory Analyses

**Fig. 3:**
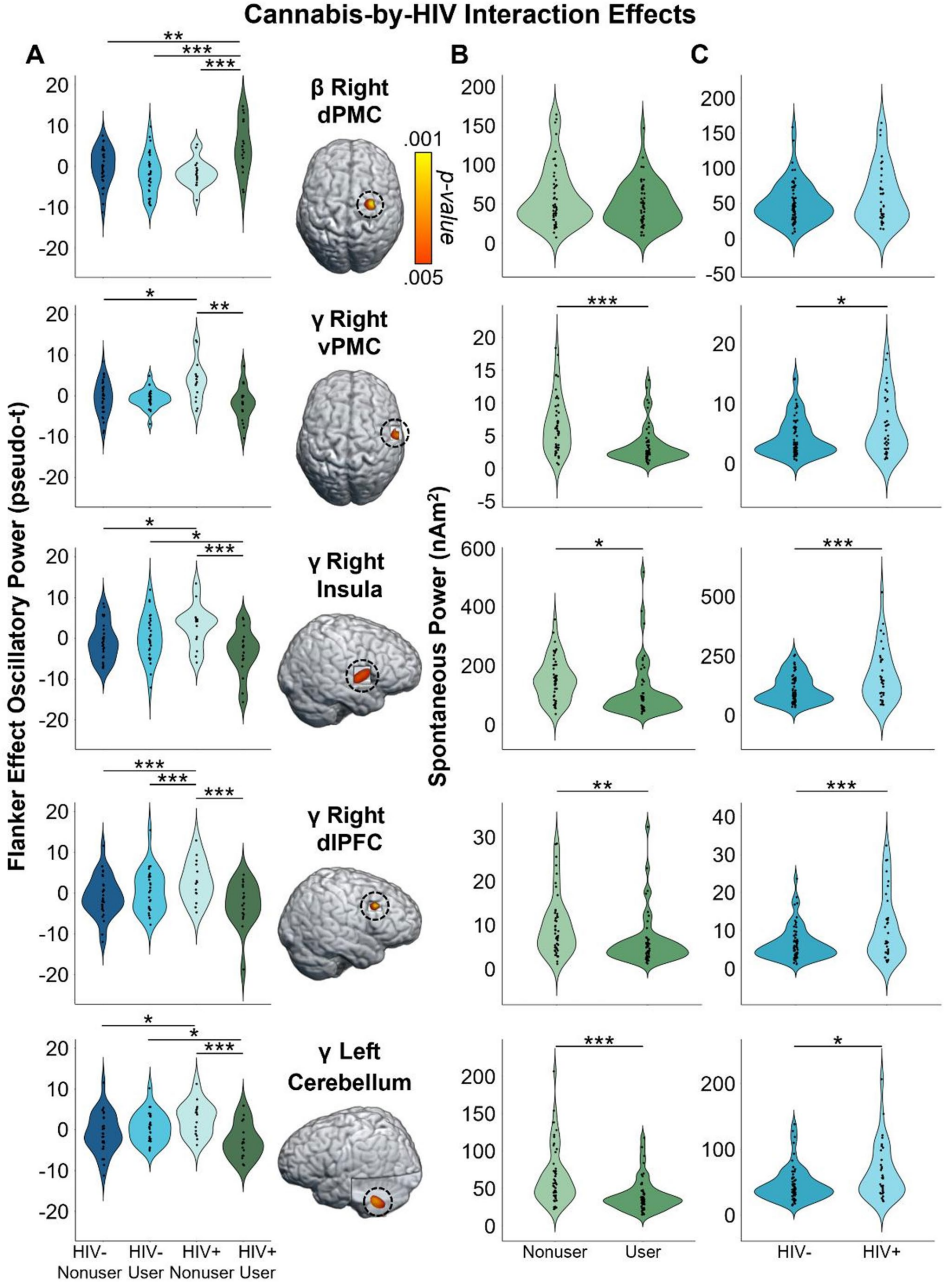
Cannabis use and HIV status interact to modulate whole-brain gamma and beta oscillatory and spontaneous power. Voxel-wise 2 × 2 ANOVAs on flanker interference maps revealed significant cannabis-by-HIV status interactions on beta power in the right dorsal premotor cortex (dPMC), and on gamma power in the right ventral premotor cortex (vPMC), right insula, right dorsolateral prefrontal cortex (dlPFC), and left cerebellum. All maps have been thresholded at *p* <.005, corrected, with the scale bar shown to the right of the brain in the top row. (**A**): Post-hoc testing revealed that cannabis using PWH exhibited weaker beta interference responses (i.e., less negative) in the right dPMC relative to each of the other three groups. Meanwhile, gamma interference responses were aberrantly elevated (i.e., more positive) in non-using PWH relative to seronegative nonusers and to cannabis-using PWH across all four cannabis-by-HIV status interaction peaks. Other group comparisons were also significant in specific regions. (**B**, **C**): We probed the spontaneous power during the baseline period (i.e., -1800 to -1000 ms) within each region that had interaction effects for oscillatory gamma or beta activity. There were significant main effects of (**B**) cannabis use and (**C**) HIV status on spontaneous gamma power in all regions such that spontaneous gamma activity was reduced in participants who use cannabis compared to non-using participants, and elevated in PWH compared to seronegative participants. *p ≤.05; **p ≤.005; ***p ≤.001

**Fig. 4:**
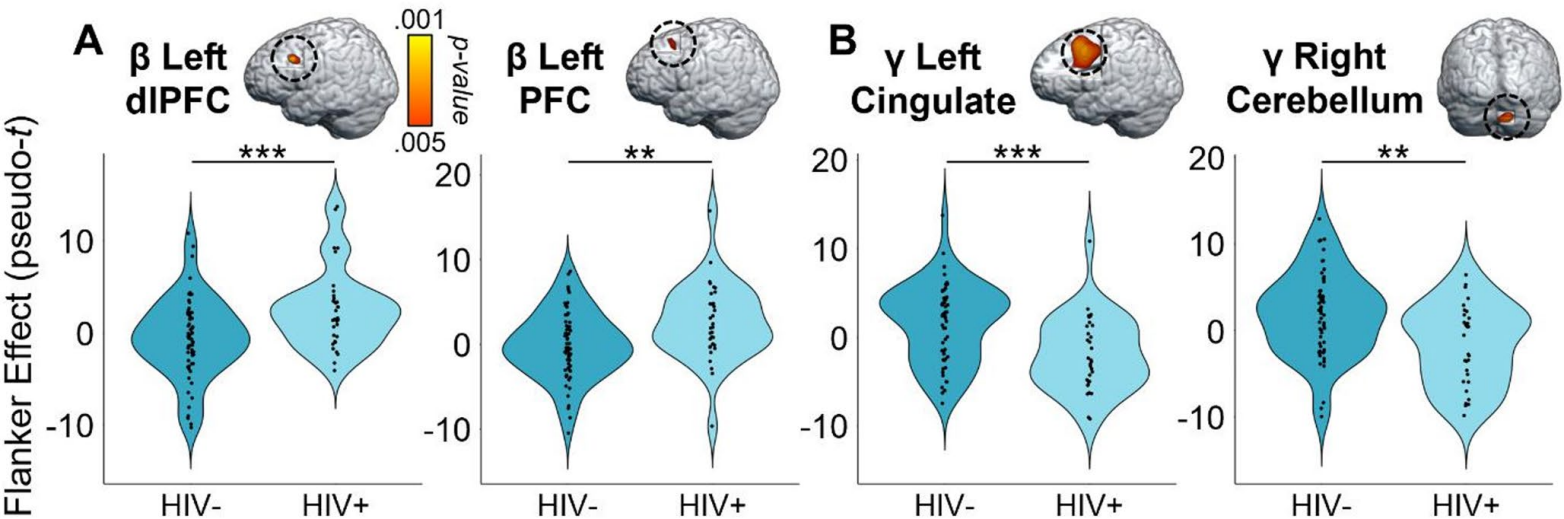
Whole-brain oscillatory beta and gamma activity differs by HIV status. Voxel-wise 2 × 2 ANOVAs revealed a significant effect of HIV status on (**A**) beta power in the left dorsolateral prefrontal cortex (dlPFC) and left prefrontal cortex (PFC), and on (**B**) gamma power in the left cingulate, right cerebellum, and left thalamus (not shown). All maps have been thresholded at *p* <.005, corrected, with the scale bar shown to the right of the first brain in the top row. Follow-up analyses revealed that, across all regions exhibiting an effect of HIV status, PWH exhibited significantly weaker flanker interference beta and gamma oscillatory power compared to controls, regardless of cannabis use status. **p ≤.005; ***p ≤.001

**Fig. 5:**
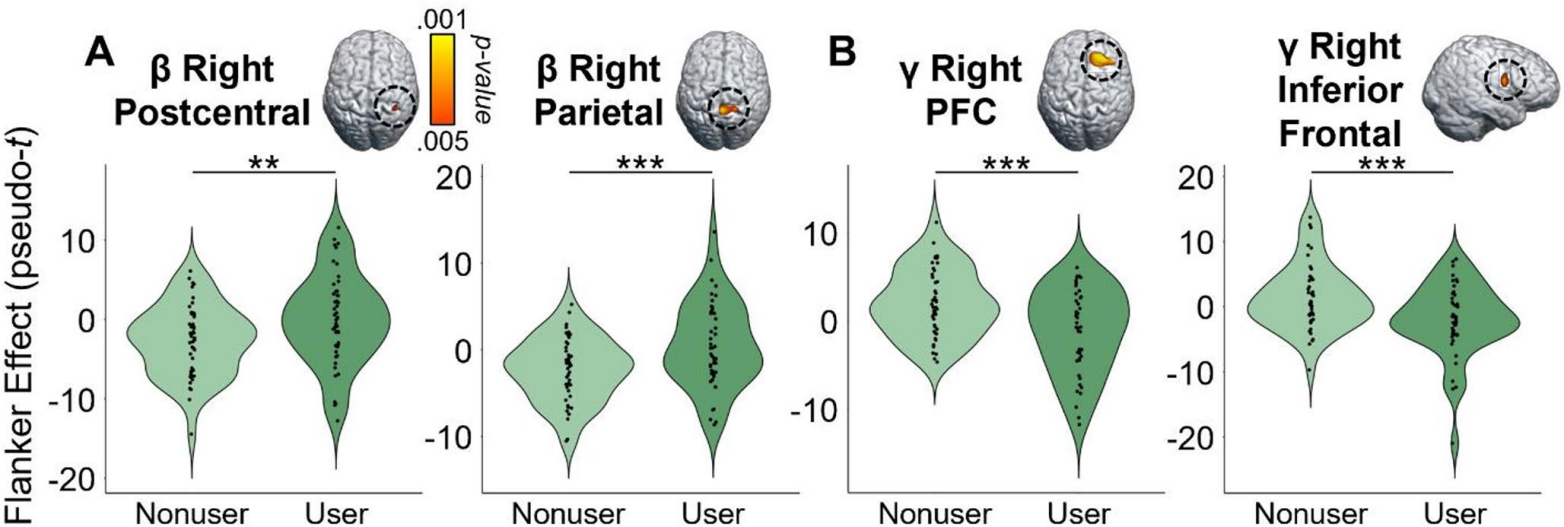
Whole-brain oscillatory beta and gamma activity differs by cannabis use. Voxel-wise 2 × 2 ANOVAs revealed a significant effect of cannabis use on (**A**) beta power in the right postcentral gyrus, parietal cortex, and ventromedial prefrontal cortex (vmPFC; not shown), and on (**B**) gamma power in the right prefrontal cortex (PFC), right cerebellum (not shown), right superior parietal cortex (not shown), and right inferior frontal gyrus. All maps have been thresholded at *p* <.005, corrected, with the scale bar shown to the right of the first brain in the top row. In general, people who regularly use cannabis exhibited significantly weaker beta and gamma interference responses compared to non-using participants, regardless of HIV status. The only exception was for gamma in the right superior parietal where the effect was reversed. **p ≤.005; ***p ≤.001

Given our focus on how the cognitive features of the task affect motor control, we first computed flanker interference maps for each time-frequency component (i.e., beta and gamma) and then conducted voxel-wise 2 × 2 (cannabis use-by-HIV status) ANOVAs. Given the trending group-wise age difference, we also re-ran these two ANOVA models as ANCOVAs with age as a covariate of no interest. All of our significant clusters remained in these ANCOVAs. For the peri-movement beta ERD statistical maps, we observed significant main effects of cannabis group and HIV status, as well as a cannabis-by-HIV group interaction effect. Specifically, the two-way interaction map had a single cluster in the right dorsal premotor cortex (dPMC; *F* = 14.50, *p* =.0003, *k* = 27) and post-hoc testing indicated that cannabis using PWH exhibited weaker beta interference responses (i.e., less negative) relative to each of the other three groups (HIV- Nonusers: *t* = 3.13, *p* =.002; HIV- Users: *t* = 4.35, *p* = 3.52 × 10^− 5^; HIV + Nonusers: *t* = 3.63, *p* =.0005; Fig. 3). The other three groups did not differ from each other, but the post-hoc tests are reported in Supplementary Table 1. Regarding the HIV main effect, clusters in the left dorsolateral prefrontal cortex (dlPFC; *F* = 11.50, *p* =.001, *k* = 26) and left prefrontal cortex (PFC; *F* = 9.45, *p* =.003, *k* = 10) were detected, with data in both regions indicating that PWH, regardless of cannabis use status, exhibited significantly weaker beta interference responses in these regions (Fig. 4A). Finally, we observed main effects of cannabis group in the right superior parietal cortices (*F* = 13.83, *p* =.0003, *k* = 75), right postcentral gyrus (*F* = 10.58, *p* =.002, *k* = 7), and the right ventromedial prefrontal cortices (vmPFC; *F* = 10.14, *p* =.002, *k* = 17), with cannabis users exhibiting significantly weaker beta interference responses in all three regions regardless of HIV status (Fig. 5A). All test statistics are reported in Supplementary Table 1.

As for gamma, we again observed significant main effects of cannabis and HIV status, as well as cannabis-by-HIV group interaction effects. Specifically, the significant interaction clusters were centered in the right ventral premotor cortex (vPMC; *F* = 10.91, *p* =.001, *k* = 41), right insula (*F* = 10.58, *p* =.002, *k* = 74), right dlPFC (*F* = 13.88, *p* =.0004, *k* = 14), and left cerebellar cortices (*F* = 12.13, *p* =.001, *k* = 178; Fig. 3). Follow-up testing of the right vPMC peak indicated that PWH who do not use cannabis exhibited stronger gamma interference responses relative to cannabis using PWH *(t* = 3.02, *p* =.003*)* and seronegative nonusers (*t* = 2.23, *p* = 028). No other post-hoc comparisons were significant, but these are listed in Supplementary Table 2. A similar pattern was observed in the right insula, as follow-up testing revealed stronger gamma interference responses in PWH who do not use cannabis compared to cannabis using PWH (*t* = 3.46, *p* =.0008) and seronegative nonusers (*t* = 2.31, *p* =.024). Seronegative cannabis users also exhibited stronger gamma interference responses in the right insula relative to cannabis using PWH (*t* = 2.27, *p* =.026). No other group comparisons were significant. The exact same pattern held in the left cerebellum, with non-using PWH exhibiting stronger gamma interference responses relative to cannabis using PWH (*t* = 3.58, *p* =.0006) and seronegative nonusers (*t* = 2.56, *p* =.012), and seronegative cannabis users having stronger gamma interference responses relative to cannabis using PWH (*t* = 2.43, *p* =.017). Finally, in the right dlPFC, follow-up testing indicated that PWH who do not use cannabis exhibited stronger gamma interference responses relative to cannabis using PWH (*t* = 4.09, *p* =.0001), seronegative nonusers *t* = 3.35, *p* =.001), and seronegative cannabis users (*t* = 3.37, *p* =.001). Regarding the main effect of HIV, significant clusters were detected in the left anterior cingulate (*F* = 18.44, *p* = 4.68 × 10^− 5^, *k* = 420), right cerebellum (*F* = 10.98, *p* =.001, *k* = 26), and a left thalamic motor area (*F* = 9.83, *p* =.002, *k* = 49). In all three regions, gamma interference responses were significantly weaker in PWH, regardless of cannabis use status (Fig. 4B). Lastly, main effects of cannabis use were significant in the right PFC (*F* = 17.23, *p* = 7.90 × 10^− 5^, *k* = 192), right superior parietal cortices (*F* = 12.49, *p* =.001, *k* = 30), right cerebellum (*F* = 15.50, *p* =.0002, *k* = 76), and the right inferior frontal gyrus (*F* = 11.37, *p* =.001, *k* = 26), with cannabis users exhibiting significantly stronger gamma interference responses in the right superior parietal cortices and significantly weaker gamma interference responses in the other three regions, regardless of HIV status (Fig. 5B). All test statistics are reported in Supplementary Table 2.

### Spontaneous Cortical Activity

Given the extensive evidence that PWH exhibit elevated spontaneous cortical activity (Spooner et al. 2018, 2020; Wiesman et al. 2018; Lew et al. 2018; Wilson et al. 2019; Casagrande et al. 2021, 2022; Christopher-Hayes et al. 2021; O’Connor et al. 2023), we probed whether such activity was altered in brain regions where cannabis-by-HIV interactions were observed. To this end, we extracted voxel time series from the peak voxels for each significant cluster exhibiting this interaction, computed the mean activity level (i.e., beta or gamma) during the pre-movement baseline period (-1800 to -1000 ms) across both task conditions, and then examined the resulting values using 2 × 2 ANOVAs (cannabis use-by-HIV status). For beta in the right dPMC, neither main effects (HIV: *F* = 2.47, *p* =.119; Cannabis: *F* = 3.27, *p* =.074) nor the interaction were significant (*F* = 1.25, *p* =.267). In contrast, all four regions that had interaction effects for gamma oscillatory activity (i.e., right vPMC, right insula, right dlPFC, and left cerebellar cortices) also exhibited main effects of both cannabis and HIV for spontaneous gamma (all *p*s < 0.005, corrected; see Table 2 for full statistics; Fig. 3, Right), but no interaction effect (Right vPMC: *F* = 0.11, *p* =.738; Right insula: *F* = 0.47, *p* =.494; Right dlPFC: *F* = 0.18, *p* =.672; Left cerebellum: *F* = 1.09, *p* =.299). Post hoc testing showed that PWH exhibited significantly elevated spontaneous gamma activity relative to seronegative participants, while those who use cannabis had significantly reduced spontaneous gamma relative to nonusers (Table 2; Fig. 3).

**Table 2:** Test statistics for 2 × 2 ANOVA on spontaneous gamma power

Effect	Region	Group Comparison		t-value	*p*-value
HIV Main Effect	Right vPMC	HIV-	HIV+	-2.64	0.010 *
	Right Insula	HIV-	HIV+	-3.37	0.001 *
	Left Cerebellum	HIV-	HIV+	-2.78	0.007 *
	Right dlPFC	HIV-	HIV+	-3.41	< 0.001 *
Cannabis Main Effect	Right vPMC	Nonuser	User	4.11	< 0.001 *
	Right Insula	Nonuser	User	2.24	0.028 *
	Left Cerebellum	Nonuser	User	4.19	< 0.001 *
	Right dlPFC	Nonuser	User	3.05	0.003 *

Note. vPMC – Ventromedial premotor cortex; dlPFC – dorsolateral prefrontal cortex; *p <.05

### Oscillatory and Spontaneous Activity in M1

**Fig. 6:**
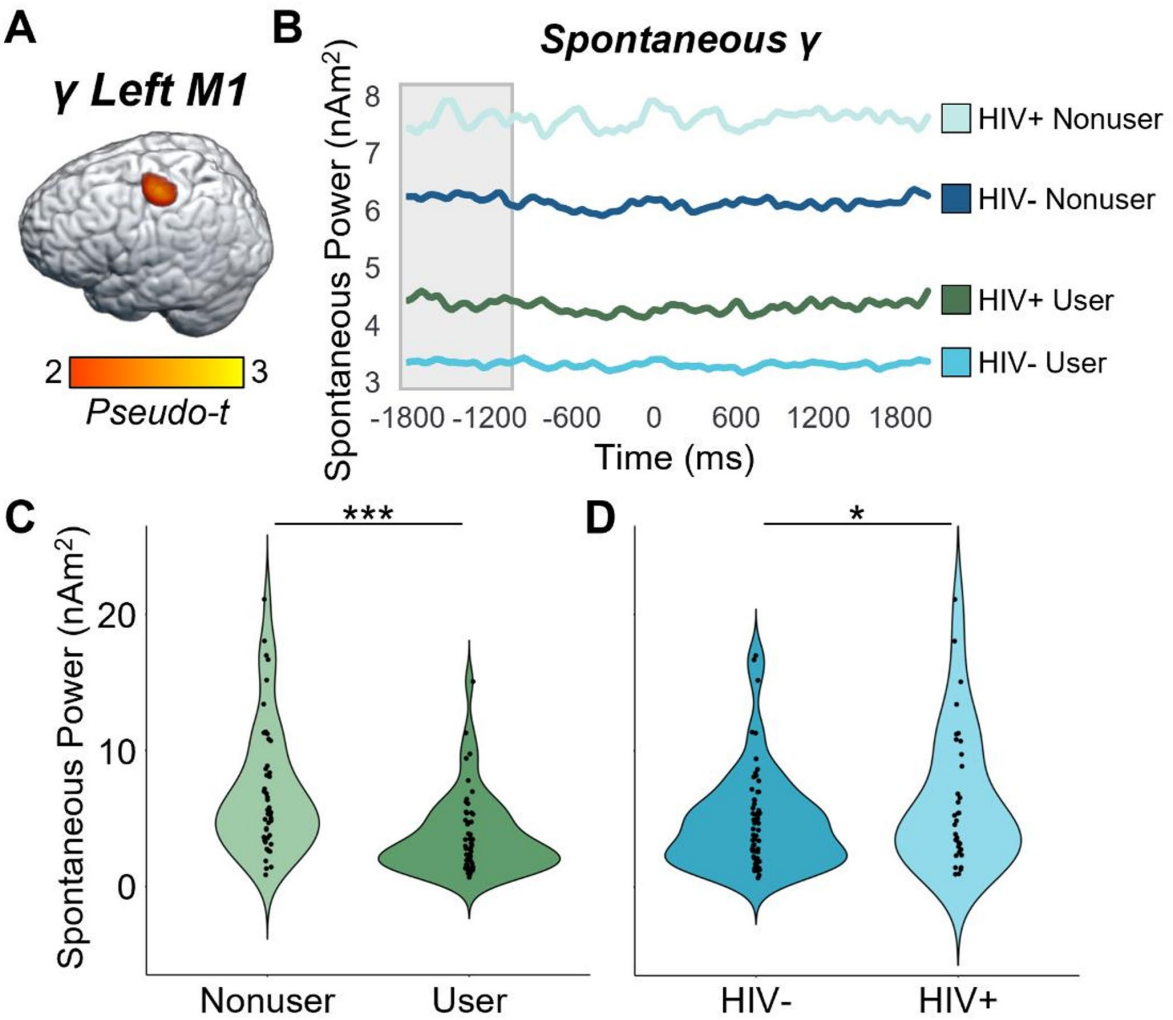
Group differences in spontaneous gamma power in the left primary motor cortex. (**A**): The grand-averaged map across both conditions and all participants indicated that the peak movement-related gamma response was centered on the motor hand knob of the left primary motor cortex (M1). The strength of the movement-related gamma oscillatory response (-75 to 75 ms) and the mean spontaneous gamma levels during the pre-movement baseline period were computed using data from the peak voxel of this cluster. (**B**): Spontaneous levels during the baseline period (-1800 to -1000 ms, gray shaded area) are plotted separately per group. (**C**): Spontaneous gamma activity was significantly weaker in chronic cannabis users relative to nonusers. (**D**): PWH exhibited significantly elevated spontaneous gamma activity relative to seronegative participants. No group differences or interactions were observed for the movement-related gamma response (-75 to 75 ms). *p ≤.05; **p ≤.005; ***p ≤.001

While our main ANOVAs did not indicate that cannabis use or HIV status affected oscillatory responses underlying the flanker interference effect in the primary motor cortices, the impact of HIV and cannabis on this crucial brain region for motor control was still of major interest. Thus, we extracted the peak voxel value from the grand-averaged oscillatory maps in Fig. 2 (i.e., the left M1 peak), computed the mean spontaneous activity level for this same voxel during the pre-movement baseline period, and then conducted 2 × 2 ANOVAs for beta and gamma activity separately. For the peri-movement beta ERD peak, neither the main effects nor the interaction were significant for the primary response (-300 to 300 ms; HIV: *F* = 0.93, *p* =.338; cannabis: *F* = 1.32, *p* =.254; HIV-by-cannabis: *F* = 0.05, *p* =.825), nor for spontaneous beta during the pre-movement baseline period (-1800 to -1000 ms; HIV: *F* = 0.61, *p* =.437; cannabis: *F* = 0.23, *p* =.635; HIV-by-cannabis: *F* = 0.09, *p* =.763). In contrast, there were main effects of cannabis (*F* = 18.26, *p* = 4.54 × 10^− 5^) and HIV (*F* = 4.10, *p* =.046) for spontaneous gamma activity during the baseline period, with PWH exhibiting elevated spontaneous gamma relative to seronegative participants and cannabis users exhibiting weaker spontaneous gamma relative to nonusers (Fig. 6). The interaction effect for spontaneous gamma was not significant (*F* = 0.50, *p* =.483) and neither the main effects nor the interaction were significant for motor-related gamma oscillations during the active window (-75 to 75 ms; HIV: *F* = 0.69, *p* =.409; cannabis: *F* = 1.39, *p* =.242; HIV-by-cannabis: F = 0.75, *p* =.390).

### Neural-Behavioral Correlations

Finally, we assessed whether spontaneous activity or the movement-related oscillatory responses within M1 and the regions exhibiting cannabis-by-HIV status interaction effects were related to the behavioral flanker effect or cannabis use metrics using Pearson bivariate correlations. These analyses indicated that spontaneous gamma activity during the baseline was positively correlated with the reaction time flanker interference effect across all participants in left M1 (*r* =.23, *p* =.022) and all four brain regions that exhibited a cannabis-by-HIV status interaction effect (Right vPMC: *r* =.35, *p* =.0004; Right anterior insula: *r* =.28, *p* =.004; Right dlPFC: *r* =.22, *p* =.026; Left cerebellum: *r* =.27, *p* =.007; Supplementary Table 3, Supplementary Fig. 1). Across all of these regions, higher spontaneous gamma (i.e., more abnormal) was associated with larger behavioral flanker interference effects (i.e., worse performance). No other correlations with spontaneous beta activity, or with oscillatory gamma or beta power, were significant, but these are listed in Supplementary Table 3. Note that using partial correlations controlling for participant age did not change the above findings.

## Discussion

In the current study, we utilized MEG to investigate the impact of regular cannabis use, HIV status, and their interaction on behavior and oscillatory neural activity underlying motor control during a cognitive interference task. Across all participants, we observed the well-known reaction time flanker effect, whereby participants responded more slowly in the presence of cognitive interference (i.e., incongruent trials) compared to the no interference condition (i.e., congruent trials). In addition, we found that PWH were more susceptible to interference (i.e., exhibited a larger flanker effect) than seronegative participants, regardless of their cannabis use status. Regarding the MEG data, all participants exhibited the well-known beta ERD and motor-related gamma oscillatory responses in each condition and the resulting data were used to compute whole-brain flanker interference maps for each response. Our key findings were cannabis use-by-HIV status interaction effects in the dorsal right PMC for beta interference activity, as well as similar interactions for gamma interference activity in the right ventral PMC, right insula, right dlPFC, and the right cerebellum. Post-hoc testing indicated that cannabis using PWH had weaker beta interference effects than all other groups in the dorsal PMC, whereas non-using PWH generally had stronger gamma interference responses than the other groups in all significant regions. Further, we found that spontaneous gamma activity in all regions exhibiting interaction effects, as well as the left primary motor cortex, was elevated in PWH, suppressed in cannabis users, and positively correlated with the behavioral flanker effect. Lastly, we found that PWH exhibited weaker beta and gamma interference effects across left prefrontal and cingulate cortices, while cannabis users generally exhibited weaker beta and gamma interference effects across a network of right hemispheric regions. We discuss the broad implications of these findings below.

Our most important findings were likely the cannabis-by-HIV status interaction effects, which suggested unique modulation of motor-related beta and gamma neural oscillations during cognitive interference. First, we found that gamma interference responses were significantly stronger in the right ventral PMC, right insula, right dlPFC, and left cerebellar cortices in non-using PWH compared to PWH who regularly use cannabis and non-using controls. Broadly, these data agree with previous work in that the cannabis using PWH exhibited gamma responses that were similar to controls, while non-using PWH exhibited more aberrant responses (Christopher-Hayes et al. 2021; Schantell et al. 2022a). Given the direction of the effect, we propose that the differences observed in the dlPFC reflect stronger engagement of this region in the suppression of interference in the non-using PWH, which may have aided in task performance. Interestingly, we found differences in a similar region in our previous study of motor control in PWH (Wilson et al. 2013), although this difference was in the beta range (gamma was not probed in the prior study). Recent work using a similar task has also shown that interference effects become stronger with increasing age in this region (Arif et al. 2024), which may be related to previous findings of accelerated aging in non-using PWH (Lew et al. 2020, 2021; Schantell et al. 2022b). Regarding the other brain regions, past studies of cognitive interference have repeatedly reported oscillatory responses in these areas (McDermott et al. 2017; Wiesman and Wilson 2020b; Wiesman et al. 2020; Arif et al. 2020), which again suggests that non-using PWH rely on stronger responses in these brain regions to maintain adequate task performance relative to PWH who use cannabis and seronegative controls. Mechanistically, we were not surprised to find interaction effects in these regions given the high density of CB1 and CB2 receptors in these regions (Bloomfield et al. 2019). THC acts as an agonist of CB1 receptors primarily found on GABAergic interneurons, which are among the primary generators of the canonical beta- and gamma-band rhythms in the brain, and CB2 receptors, which are most abundant on immune cells and neurons and are thought to reflect anti-inflammatory processes (Lu and Mackie 2021). Beyond gamma activity, we also found that beta interference responses in the right dorsal PMC were weaker in cannabis using PWH than all other groups. These data may indicate that cannabis has a unique impact on neural oscillations in this brain region among those with HIV, but more work is needed. Of note, beta interference responses in this brain region have been reported in previous normative studies (Wiesman et al. 2020), which suggests at least some functional role in suppressing interference during motor performance.

A second area of major new findings surrounds our spontaneous cortical activity data. While we did not find differences in spontaneous beta, we observed a consistent pattern of elevated spontaneous gamma in PWH and suppressed spontaneous gamma power in those who regularly use cannabis. This pattern was observed across all four regions (i.e., right ventral PMC, right insula, right dlPFC, and left cerebellum) where we observed gamma-based interaction effects in the main analysis, as well as the left primary motor cortex. Furthermore, in all of these regions, the strength of spontaneous gamma activity was positively correlated with the behavioral reaction time flanker effect, suggesting that those with the most elevated spontaneous gamma were the most susceptible to interference (i.e., performed the worst). These findings corroborate previous literature also reporting aberrantly-elevated spontaneous neural activity in PWH (Spooner et al. 2018, 2020; Wiesman et al. 2018; Lew et al. 2018; Casagrande et al. 2021; Christopher-Hayes et al. 2021), which in some cases has also distinguished cognitively impaired PWH from unimpaired PWH (Wiesman et al. 2018; Lew et al. 2018; Spooner et al. 2020) and PWH from those with Alzheimer’s disease (Casagrande et al. 2022). Second, our finding that spontaneous gamma activity was suppressed in participants who regularly use cannabis agrees with numerous recent studies (Arif et al. 2021; Weyrich et al. 2023; Schantell et al. 2024; Webert et al. 2024). Given the widespread nature of this effect, we propose it may be linked to the mechanistic action of cannabis on endocannabinoid 1 receptors (CB1), which are G-protein coupled receptors that are known to be located on neurons throughout the cortex (Lu and Mackie 2021). Importantly, at least two previous studies have shown that cannabis use has a normalizing effect on the aberrant HIV-related elevation in spontaneous activity (Christopher‐Hayes et al. 2021; Schantell et al. 2022a). Thus, the current study expands on these findings by showing that the effect is clearly not specific to PWH but is more general and affects all cannabis users. However, since PWH exhibit elevated levels of spontaneous gamma activity in the absence of cannabis, the net effect is a normalization of such activity, whereas in seronegative controls the net impact is spontaneous activity being sharply below that of non-using healthy adults. Our finding that some brain responses in PWH are normalized by regular cannabis use is a major contribution to the field. While the full implications of these findings remain unclear, they may be associated with declines in local oscillatory function, as well as a suppression of aberrant neuroinflammation in PWH (Manuzak et al. 2018; Watson et al. 2021). This is only the third study to our knowledge to show this effect, though it is promising in the domain of pharmaco-neurotherapeutic approaches to address HIV-related inflammation and cognitive decline, particularly given the anti-inflammatory properties of cannabis (Manuzak et al. 2018; Ellis et al. 2020). Future work should continue to pursue this line, especially given the findings from prior work that spontaneous activity levels can distinguish cognitively impaired from unimpaired PWH and both groups from controls (Wiesman et al. 2018; Lew et al. 2018; Spooner et al. 2020), as well as PWH from those with Alzheimer’s disease spectrum conditions (Casagrande et al. 2022).

We also observed main effects of cannabis use and HIV status in several brain regions, and these were found for both beta and gamma oscillations. Main effects of cannabis use were concentrated in the right hemisphere, especially the prefrontal and parietal cortices, and included weaker beta interference responses in the right superior parietal, right postcentral gyrus, and right vmPFC, as well as weaker gamma interference responses in the right PFC, right cerebellum, and right inferior frontal gyrus of the cannabis users relative to nonusers. Notably, the opposite pattern of stronger gamma interference responses was found in the right superior parietal cortices of users. Regarding the right hemispheric bias, this could simply reflect the greater involvement of parietal and prefrontal regions in the dorsal attention network of the nonusers, as the flanker interference task is known to have a major selective attention component (Wilson et al. 2016; Taylor et al. 2021). The specific regions involved would support this interpretation due to their role in the cognitive control of movement and attention (Ito 2005; Cieslik et al. 2015; Hardwick et al. 2015; Rangel-Pacheco et al. 2021), as well as the regions exhibiting high CB1 and CB2 receptor densities (Bloomfield et al. 2019; Lu and Mackie 2021). In contrast, the HIV status main effects tended to concentrate in the left hemisphere, with weaker beta interference responses in PWH in the left dlPFC and left PFC, as well as weaker gamma interference responses in the left ACC, right cerebellum, and left thalamic motor area of PWH relative to seronegative participants. Such a bias toward greater HIV-related aberrations in left prefrontal and ACC regions relative to their right hemispheric homologues is consistent with numerous reports (Wilson et al. 2017, 2019; Lew et al. 2018; Spooner et al. 2020; Arif et al. 2020), although the mechanisms remain poorly understood and additional studies are certainly warranted.

Before closing, it is important to note the limitations of the current study. First, there is no sophisticated approach in the literature to account for differences between cannabis intake modalities and potency of different strains. Thus, it is possible that differences in tetrahydrocannabinol (THC) concentration (i.e., the main psychoactive component in cannabis) could have impacted our results by increasing variances within the cannabis groups. While this is certainly possible, we do not believe it would have a major impact on the conclusions of the study, especially since the two cannabis groups are unlikely to systematically differ from each other on these parameters. A second limitation of the study is that the button response to the flanker task is a contrived motor response. Future work should utilize motor tasks that allow for more naturalistic movements to investigate the neural dynamics underling motor control. A third limitation of the present study is the inability to generalize our results to PWH who are not virally suppressed. This was part of the inclusion criteria for the current study, and we would hypothesize that the group differences would be much larger in those who were not virally suppressed, given the elevated levels of sustained inflammation. Future work should consider directly probing this line of inquiry. Despite these limitations, the current study found evidence of multiple novel interactions between cannabis use and HIV status in beta and gamma interference responses across a broad network of brain regions. Further, these findings corroborate multiple recent studies showing elevated spontaneous gamma activity in PWH, and that regular cannabis use is associated with a marked suppression in such spontaneous activity. Future work is needed to decipher whether suppressing pathologically elevated spontaneous gamma activity with cannabis in PWH has a net positive effect on cognitive and brain health.

## Electronic Supplementary Material

Below is the link to the electronic supplementary material.

Supplementary Material 1

## Data Availability

De-identified data have been made available to the public through the Collaborative Informatics and Neuroimaging Suite (COINS) database.

## References

[CR1] Antinori A, Arendt G, Becker JT et al (2007) Updated research nosology for HIV-associated neurocognitive disorders. Neurology 69:1789–1799. 10.1212/01.WNL.0000287431.88658.8b 17914061 10.1212/01.WNL.0000287431.88658.8b PMC4472366

[CR4] Arif Y, Wiesman AI, O’Neill J et al (2020) The age-related trajectory of visual attention neural function is altered in adults living with HIV: A cross-sectional MEG study. EBioMedicine 61:103065. 10.1016/j.ebiom.2020.103065 33099087 10.1016/j.ebiom.2020.103065 PMC7585051

[CR3] Arif Y, Wiesman AI, Christopher-Hayes NJ, Wilson TW (2021) Aberrant inhibitory processing in the somatosensory cortices of cannabis-users. J Psychopharmacol (Oxf) 35:1356–1364. 10.1177/02698811211050557 10.1177/02698811211050557 PMC9659470 34694190

[CR2] Arif Y, Son JJ, Okelberry HJ et al (2024) Modulation of movement-related oscillatory signatures by cognitive interference in healthy aging. GeroScience 46:3021–3034. 10.1007/s11357-023-01057-0 38175521 10.1007/s11357-023-01057-0 PMC11009213

[CR5] Becker KM, Heinrichs-Graham E, Fox HS et al (2013) Decreased MEG beta oscillations in HIV-infected older adults during the resting state. J Neurovirol 19:586–594. 10.1007/s13365-013-0220-8 24297500 10.1007/s13365-013-0220-8 PMC3913174

[CR6] Bloomfield MAP, Hindocha C, Green SF et al (2019) The neuropsychopharmacology of cannabis: A review of human imaging studies. Pharmacol Ther 195:132–161. 10.1016/j.pharmthera.2018.10.006 30347211 10.1016/j.pharmthera.2018.10.006 PMC6416743

[CR7] Casagrande CC, Lew BJ, Taylor BK et al (2021) Impact of HIV-infection on human somatosensory processing, spontaneous cortical activity, and cortical thickness: A multimodal neuroimaging approach. Hum Brain Mapp 42:2851–2861. 10.1002/hbm.25408 33738895 10.1002/hbm.25408 PMC8127147

[CR8] Casagrande CC, Wiesman AI, Schantell M et al (2022) Signatures of somatosensory cortical dysfunction in Alzheimer’s disease and HIV-associated neurocognitive disorder. Brain Commun 4:fcac169. 10.1093/braincomms/fcac169 35813878 10.1093/braincomms/fcac169 PMC9260304

[CR9] Cheyne D, Bells S, Ferrari P et al (2008) Self-paced movements induce high-frequency gamma oscillations in primary motor cortex. NeuroImage 42:332–342. 10.1016/j.neuroimage.2008.04.178 18511304 10.1016/j.neuroimage.2008.04.178

[CR10] Chou S, Ranganath T, Fish KN et al (2022) Cell type specific cannabinoid CB1 receptor distribution across the human and non-human primate cortex. Sci Rep 12:9605. 10.1038/s41598-022-13724-x 35688916 10.1038/s41598-022-13724-x PMC9187707

[CR11] Christopher-Hayes NJ, Lew BJ, Wiesman AI et al (2021) Cannabis use impacts pre‐stimulus neural activity in the visual cortices of people with HIV. Hum Brain Mapp 42:5446–5457. 10.1002/hbm.25634 34464488 10.1002/hbm.25634 PMC8519863

[CR12] Cieslik EC, Mueller VI, Eickhoff CR et al (2015) Three key regions for supervisory attentional control: evidence from neuroimaging meta-analyses. Neurosci Biobehav Rev 48:22–34. 10.1016/j.neubiorev.2014.11.003 25446951 10.1016/j.neubiorev.2014.11.003 PMC4272620

[CR14] Ellis RJ, Peterson SN, Li Y et al (2020) Recent cannabis use in HIV is associated with reduced inflammatory markers in CSF and blood. Neurol - Neuroimmunol Neuroinflammation 7:e809. 10.1212/NXI.0000000000000809 10.1212/NXI.0000000000000809 PMC7309527 32554630

[CR13] Ellis RJ, Marquine MJ, Kaul M et al (2023) Mechanisms underlying HIV-associated cognitive impairment and emerging therapies for its management. Nat Rev Neurol 19:668–687. 10.1038/s41582-023-00879-y 37816937 10.1038/s41582-023-00879-y PMC11052664

[CR15] Ernst MD (2004) Permutation methods: A basis for exact inference. Stat Sci 19. 10.1214/088342304000000396

[CR16] Groff BR, Wiesman AI, Rezich MT et al (2020) Age-related visual dynamics in HIV-infected adults with cognitive impairment. Neurol - Neuroimmunol Neuroinflammation 7:e690. 10.1212/NXI.0000000000000690 10.1212/NXI.0000000000000690 PMC7051212 32102916

[CR17] Gross J, Kujala J, Hämäläinen M et al (2001) Dynamic imaging of coherent sources: studying neural interactions in the human brain. Proc Natl Acad Sci 98:694–699. 10.1073/pnas.98.2.694 11209067 10.1073/pnas.98.2.694 PMC14650

[CR18] Hardwick RM, Lesage E, Eickhoff CR et al (2015) Multimodal connectivity of motor learning-related dorsal premotor cortex. NeuroImage 123:114–128. 10.1016/j.neuroimage.2015.08.024 26282855 10.1016/j.neuroimage.2015.08.024 PMC4780681

[CR20] Heaton RK, Miller SW, Taylor MJ, Grant I (2004) Revised comprehensive norms for an expanded Halstead-Reitan battery: demographically adjusted neuropsychological norms for African American and Caucasian adults. Lutz FL Psychol Assess Resour

[CR19] Heaton RK, Franklin DR, Ellis RJ et al (2011) HIV-associated neurocognitive disorders before and during the era of combination antiretroviral therapy: differences in rates, nature, and predictors. J Neurovirol 17:3–16. 10.1007/s13365-010-0006-1 21174240 10.1007/s13365-010-0006-1 PMC3032197

[CR23] Heinrichs-Graham E, Wilson TW (2015) Coding complexity in the human motor circuit. Hum Brain Mapp 36:5155–5167. 10.1002/hbm.23000 26406479 10.1002/hbm.23000 PMC4715608

[CR22] Heinrichs-Graham E, Kurz MJ, Gehringer JE, Wilson TW (2017) The functional role of post-movement beta oscillations in motor termination. Brain Struct Funct 222:3075–3086. 10.1007/s00429-017-1387-1 28337597 10.1007/s00429-017-1387-1 PMC5610915

[CR21] Heinrichs-Graham E, Hoburg JM, Wilson TW (2018) The peak frequency of motor-related gamma oscillations is modulated by response competition. NeuroImage 165:27–34. 10.1016/j.neuroimage.2017.09.059 28966082 10.1016/j.neuroimage.2017.09.059 PMC5826720

[CR24] Ito M (2005) Bases and implications of learning in the cerebellum — adaptive control and internal model mechanism. Progress in brain research. Elsevier, pp 95–109 10.1016/S0079-6123(04)48009-1 15661184

[CR25] Kovach CK, Gander PE (2016) The demodulated band transform. J Neurosci Methods 261:135–154. 10.1016/j.jneumeth.2015.12.004 26711370 10.1016/j.jneumeth.2015.12.004 PMC5084918

[CR26] Lawton MP, Brody EM (1969) Assessment of older people: Self-Maintaining and instrumental activities of daily living. Gerontologist 9:179–186. 10.1093/geront/9.3_Part_1.179 5349366

[CR27] Lew BJ, McDermott TJ, Wiesman AI et al (2018) Neural dynamics of selective attention deficits in HIV-associated neurocognitive disorder. Neurology 91:e1860–e1869. 10.1212/WNL.0000000000006504 30333162 10.1212/WNL.0000000000006504 PMC6260195

[CR28] Lew BJ, O’Neill J, Rezich MT et al (2020) Interactive effects of HIV and ageing on neural oscillations: independence from neuropsychological performance. Brain Commun 2:fcaa015. 10.1093/braincomms/fcaa015 32322820 10.1093/braincomms/fcaa015 PMC7158235

[CR29] Lew BJ, Schantell MD, O’Neill J et al (2021) Reductions in Gray matter linked to epigenetic HIV-Associated accelerated aging. Cereb Cortex 31:3752–3763. 10.1093/cercor/bhab045 33822880 10.1093/cercor/bhab045 PMC8258439

[CR30] Lu H-C, Mackie K (2021) Review of the endocannabinoid system. Biol Psychiatry Cogn Neurosci Neuroimaging 6:607–615. 10.1016/j.bpsc.2020.07.016 32980261 10.1016/j.bpsc.2020.07.016 PMC7855189

[CR31] Manuzak JA, Gott TM, Kirkwood JS et al (2018) Heavy Cannabis use associated with reduction in activated and inflammatory immune cell frequencies in antiretroviral Therapy–Treated human immunodeficiency Virus–Infected individuals. Clin Infect Dis 66:1872–1882. 10.1093/cid/cix1116 29471387 10.1093/cid/cix1116 PMC6248381

[CR32] Maris E, Oostenveld R (2007) Nonparametric statistical testing of EEG- and MEG-data. J Neurosci Methods 164:177–190. 10.1016/j.jneumeth.2007.03.024 17517438 10.1016/j.jneumeth.2007.03.024

[CR33] McDermott TJ, Wiesman AI, Proskovec AL et al (2017) Spatiotemporal oscillatory dynamics of visual selective attention during a flanker task. NeuroImage 156:277–285. 10.1016/j.neuroimage.2017.05.014 28501539 10.1016/j.neuroimage.2017.05.014 PMC5548621

[CR34] Meehan CE, Schantell M, Wiesman AI et al (2023) Oscillatory markers of neuroHIV-related cognitive impairment and Alzheimer’s disease during attentional interference processing. Aging 15:524–541. 10.18632/aging.204496 36656738 10.18632/aging.204496 PMC9925679

[CR35] O’Connor EE, Sullivan EV, Chang L et al (2023) Imaging of brain structural and functional effects in people with human immunodeficiency virus. J Infect Dis 227:S16–S29. 10.1093/infdis/jiac387 36930637 10.1093/infdis/jiac387 PMC10022717

[CR36] Poline J-B, Worsley KJ, Holmes AP et al (1995) Estimating smoothness in statistical parametric maps: variability of P values. J Comput Assist Tomogr 19:788–796. 10.1097/00004728-199509000-00017 7560327 10.1097/00004728-199509000-00017

[CR37] Proskovec AL, Heinrichs-Graham E, Wiesman AI et al (2018) Oscillatory dynamics in the dorsal and ventral attention networks during the reorienting of attention. Hum Brain Mapp 39:2177–2190. 10.1002/hbm.23997 29411471 10.1002/hbm.23997 PMC5895484

[CR38] Purohit V, Rapaka RS, Rutter J (2014) Cannabinoid Receptor-2 and HIV-Associated neurocognitive disorders. J Neuroimmune Pharmacol 9:447–453. 10.1007/s11481-014-9554-0 25015040 10.1007/s11481-014-9554-0

[CR39] Rangel-Pacheco A, Lew BJ, Schantell MD et al (2021) Altered fronto-occipital connectivity during visual selective attention in regular cannabis users. Psychopharmacology 238:1351–1361. 10.1007/s00213-020-05717-3 33241479 10.1007/s00213-020-05717-3 PMC8068572

[CR41] Schantell M, Springer SD, Arif Y et al (2022a) Regular cannabis use modulates the impact of HIV on the neural dynamics serving cognitive control. J Psychopharmacol (Oxf) 36:1324–1337. 10.1177/02698811221138934 10.1177/02698811221138934 PMC9835727 36416285

[CR42] Schantell M, Taylor BK, Spooner RK et al (2022b) Epigenetic aging is associated with aberrant neural oscillatory dynamics serving visuospatial processing in people with HIV. Aging 14:9818–9831. 10.18632/aging.204437 36534452 10.18632/aging.204437 PMC9831734

[CR40] Schantell M, John JA, Coutant AT et al (2024) Chronic cannabis use alters the spontaneous and oscillatory gamma dynamics serving cognitive control. Hum Brain Mapp 45:e26787. 10.1002/hbm.26787 39023178 10.1002/hbm.26787 PMC11256138

[CR43] Son JJ, Arif Y, Okelberry HJ et al (2024) Aging modulates the impact of cognitive interference subtypes on dynamic connectivity across a distributed motor network. Npj Aging 10:54. 10.1038/s41514-024-00182-0 39580466 10.1038/s41514-024-00182-0 PMC11585575

[CR52] Spooner RK, Wilson TW (2022) Cortical theta–gamma coupling governs the adaptive control of motor commands. Brain Commun 4:fcac249. 10.1093/braincomms/fcac249 36337344 10.1093/braincomms/fcac249 PMC9631971

[CR53] Spooner RK, Wilson TW (2023) Spectral specificity of gamma-frequency transcranial alternating current stimulation over motor cortex during sequential movements. Cereb Cortex 33:5347–5360. 10.1093/cercor/bhac423 36368895 10.1093/cercor/bhac423 PMC10152093

[CR49] Spooner RK, Wiesman AI, Mills MS et al (2018) Aberrant oscillatory dynamics during somatosensory processing in HIV-infected adults. NeuroImage Clin 20:85–91. 10.1016/j.nicl.2018.07.009 30094159 10.1016/j.nicl.2018.07.009 PMC6070689

[CR51] Spooner RK, Wiesman AI, Proskovec AL et al (2019) Rhythmic spontaneous activity mediates the Age-Related decline in somatosensory function. Cereb Cortex 29:680–688. 10.1093/cercor/bhx349 29342238 10.1093/cercor/bhx349 PMC6319169

[CR50] Spooner RK, Wiesman AI, O’Neill J et al (2020) Prefrontal gating of sensory input differentiates cognitively impaired and unimpaired aging adults with HIV. Brain Commun 2:fcaa080. 10.1093/braincomms/fcaa080 32954330 10.1093/braincomms/fcaa080 PMC7472908

[CR44] Spooner RK, Arif Y, Taylor BK, Wilson TW (2021a) Movement-Related gamma synchrony differentially predicts behavior in the presence of visual interference across the lifespan. Cereb Cortex 31:5056–5066. 10.1093/cercor/bhab141 34115110 10.1093/cercor/bhab141 PMC8491684

[CR47] Spooner RK, Taylor BK, Ahmad IM et al (2021b) Neural oscillatory activity serving sensorimotor control is predicted by superoxide-sensitive mitochondrial redox environments. Proc Natl Acad Sci 118:e2104569118. 10.1073/pnas.2104569118 34686594 10.1073/pnas.2104569118 PMC8639326

[CR48] Spooner RK, Taylor BK, Moshfegh CM et al (2021c) Neuroinflammatory profiles regulated by the redox environment predicted cognitive dysfunction in people living with HIV: A cross-sectional study. EBioMedicine 70:103487. 10.1016/j.ebiom.2021.103487 34280780 10.1016/j.ebiom.2021.103487 PMC8318860

[CR45] Spooner RK, Taylor BK, Ahmad IM et al (2023) Mitochondrial redox environments predict sensorimotor brain-behavior dynamics in adults with HIV. Brain Behav Immun 107:265–275. 10.1016/j.bbi.2022.10.004 36272499 10.1016/j.bbi.2022.10.004 PMC10590193

[CR46] Spooner RK, Taylor BK, Ahmad IM et al (2024) Clinical markers of HIV predict redox-regulated neural and behavioral function in the sensorimotor system. Free Radic Biol Med 212:322–329. 10.1016/j.freeradbiomed.2023.12.027 38142954 10.1016/j.freeradbiomed.2023.12.027 PMC11161132

[CR54] Springer SD, Spooner RK, Schantell M et al (2023) Regular recreational Cannabis users exhibit altered neural oscillatory dynamics during attention reorientation. Psychol Med 53:1205–1214. 10.1017/S0033291721002671 34889178 10.1017/S0033291721002671 PMC9250753

[CR55] Taylor BK, Eastman JA, Frenzel MR et al (2021) Neural oscillations underlying selective attention follow sexually divergent developmental trajectories during adolescence. Dev Cogn Neurosci 49:100961. 10.1016/j.dcn.2021.100961 33984667 10.1016/j.dcn.2021.100961 PMC8131898

[CR56] Thomas JB, Brier MR, Snyder AZ et al (2013) Pathways to neurodegeneration: effects of HIV and aging on resting-state functional connectivity. Neurology 80:1186–1193. 10.1212/WNL.0b013e318288792b 23446675 10.1212/WNL.0b013e318288792b PMC3691785

[CR57] Watson CW-M, Campbell LM, Sun-Suslow N et al (2021) Daily Cannabis use is associated with lower CNS inflammation in people with HIV. J Int Neuropsychol Soc 27:661–672. 10.1017/S1355617720001447 34261550 10.1017/S1355617720001447 PMC8288448

[CR58] Webert LK, Schantell M, John JA et al (2024) Regular cannabis use modulates gamma activity in brain regions serving motor control. J Psychopharmacol (Oxf) 02698811241268876. 10.1177/02698811241268876 10.1177/02698811241268876 PMC11524774 39140179

[CR59] Weyrich L, Arif Y, Schantell M et al (2023) Altered functional connectivity and oscillatory dynamics in polysubstance and cannabis only users during visuospatial processing. Psychopharmacology 240:769–783. 10.1007/s00213-023-06318-6 36752815 10.1007/s00213-023-06318-6 PMC10545949

[CR64] Wiesman AI, Wilson TW (2020a) Attention modulates the gating of primary somatosensory oscillations. NeuroImage 211:116610. 10.1016/j.neuroimage.2020.116610 32044438 10.1016/j.neuroimage.2020.116610 PMC7111587

[CR65] Wiesman AI, Wilson TW (2020b) Posterior alpha and gamma oscillations index divergent and superadditive effects of cognitive interference. Cereb Cortex 30:1931–1945. 10.1093/cercor/bhz214 31711121 10.1093/cercor/bhz214 PMC7132948

[CR63] Wiesman AI, O’Neill J, Mills MS et al (2018) Aberrant occipital dynamics differentiate HIV-infected patients with and without cognitive impairment. Brain 141:1678–1690. 10.1093/brain/awy097 29672678 10.1093/brain/awy097 PMC5972635

[CR62] Wiesman AI, Koshy SM, Heinrichs-Graham E, Wilson TW (2020) Beta and gamma oscillations index cognitive interference effects across a distributed motor network. NeuroImage 213:116747. 10.1016/j.neuroimage.2020.116747 32179103 10.1016/j.neuroimage.2020.116747 PMC7231968

[CR60] Wiesman AI, Christopher-Hayes NJ, Eastman JA et al (2021a) Response certainty during bimanual movements reduces gamma oscillations in primary motor cortex. NeuroImage 224:117448. 10.1016/j.neuroimage.2020.117448 33059048 10.1016/j.neuroimage.2020.117448 PMC7994913

[CR61] Wiesman AI, Christopher-Hayes NJ, Wilson TW (2021b) Stairway to memory: Left-hemispheric alpha dynamics index the progressive loading of items into a short-term store. NeuroImage 235:118024. 10.1016/j.neuroimage.2021.118024 33836267 10.1016/j.neuroimage.2021.118024 PMC8354033

[CR67] Wilson TW, Heinrichs-Graham E, Robertson KR et al (2013) Functional brain abnormalities during Finger-Tapping in HIV-Infected older adults: A magnetoencephalography study. J Neuroimmune Pharmacol 8:965–974. 10.1007/s11481-013-9477-1 23749418 10.1007/s11481-013-9477-1 PMC3809128

[CR66] Wilson TW, Heinrichs-Graham E, Proskovec AL, McDermott TJ (2016) Neuroimaging with magnetoencephalography: A dynamic view of brain pathophysiology. Transl Res 175:17–36. 10.1016/j.trsl.2016.01.007 26874219 10.1016/j.trsl.2016.01.007 PMC4959997

[CR69] Wilson TW, Proskovec AL, Heinrichs-Graham E et al (2017) Aberrant neuronal dynamics during working memory operations in the aging HIV-Infected brain. Sci Rep 7:41568. 10.1038/srep41568 28155864 10.1038/srep41568 PMC5290733

[CR68] Wilson TW, Lew BJ, Spooner RK et al (2019) Aberrant brain dynamics in neurohiv: evidence from Magnetoencephalographic (MEG) imaging. Progress in molecular biology and translational science. Elsevier, pp 285–320 10.1016/bs.pmbts.2019.04.008 PMC8026013 31481167

[CR70] Winston A, Spudich S (2020) Cognitive disorders in people living with HIV. Lancet HIV 7:e504–e513. 10.1016/S2352-3018(20)30107-7 32621876 10.1016/S2352-3018(20)30107-7

[CR72] Worsley KJ, Marrett S, Neelin P et al (1996) A unified statistical approach for determining significant signals in images of cerebral activation. Hum Brain Mapp 4:58–73. 10.1002/(SICI)1097-0193(1996)4:1%3C58::AID-HBM4%3E3.0.CO;2-O 20408186 10.1002/(SICI)1097-0193(1996)4:1<58::AID-HBM4>3.0.CO;2-O

[CR71] Worsley KJ, Andermann M, Koulis T et al (1999) Detecting changes in nonisotropic images. Hum Brain Mapp 8:98–101. 10.1002/(SICI)1097-0193(1999)8:2/3%3C98::AID-HBM5%3E3.0.CO;2-F 10524599 10.1002/(SICI)1097-0193(1999)8:2/3<98::AID-HBM5>3.0.CO;2-F PMC6873343

[CR73] Zhou Y, Li R, Wang X et al (2017) Motor-related brain abnormalities in HIV-infected patients: a multimodal MRI study. Neuroradiology 59:1133–1142. 10.1007/s00234-017-1912-1 28889255 10.1007/s00234-017-1912-1

