# Supplementary material for: Motor-Related Neural Dynamics are Modulated by Regular Cannabis Use Among People with HIV
Source: J Neuroimmune Pharmacol. 2025 Jun 6;20(1):63. doi: 10.1007/s11481-025-10219-0 (PMC12141125; doi:10.1007/s11481-025-10219-0)
Supplement: Supplementary file 1 — Supplementary Material 1 [file 11481_2025_10219_MOESM1_ESM.docx]

***Supplemental Materials:***

***Title: Motor-related neural dynamics are modulated by regular cannabis use among people with HIV***

Lauren K. Webert, Mikki Schantell, Lucy K. Horne, Jason A. John, Ryan Glesinger, Jennifer O’Neill, Maureen Kubat, Anna T. Coutant, Grace C. Ende, Sara H. Bares, Pamela E. May-Weeks, Tony W. Wilson

**Supplementary Results**

*Whole-Brain Oscillatory Results*

**Supplementary Table 1.** Follow-up Statistical Tests on Oscillatory Beta ERD 2x2 ANOVA

| **Oscillatory β Effect** | Region | Group Comparison | | *t* | *p* |
| --- | --- | --- | --- | --- | --- |
| ***HIV status-by-Cannabis Interaction*** | Right dPMC | HIV- Nonuser | HIV- User | 1.43 | .157 |
|  |  |  | HIV+ Nonuser | 1.04 | .301 |
|  |  |  | HIV+ User | -3.133 | .002 * |
|  |  | HIV- User | HIV+ Nonuser | -.13 | .897 |
|  |  |  | HIV+ User | -4.35 | < .001 * |
|  |  | HIV+ Nonuser | HIV+ User | -3.63 | < .001 * |
| ***HIV Main Effect*** | Left dlPFC | HIV- | HIV+ | -3.39 | .001 * |
|  | Left PFC | HIV- | HIV+ | -3.07 | .003 * |
| ***Cannabis Main Effect*** | Right Parietal Cortex | Nonuser | User | -3.74 | < .001 * |
|  | Right Postcentral Gyrus | Nonuser | User | -3.18 | .002 * |
|  | Right vmPFC | Nonuser | User | -3.32 | .001 * |

*Note*. dPMC – Dorsal premotor cortex; dlPFC – dorsolateral prefrontal cortex; PFC – prefrontal cortex; vmPFC – ventromedial prefrontal cortex; **p* < .05.

**Supplementary Table 2.** Follow-up Statistical Tests on Oscillatory Gamma 2x2 ANOVA

| **Oscillatory γ Effect** | Region | Group Comparison | | *t* | *p* |
| --- | --- | --- | --- | --- | --- |
| ***HIV status-by-Cannabis Interaction*** | Right vPMC | HIV- Nonuser | HIV- User | -.87 | .385 |
|  |  |  | HIV+ Nonuser | -2.23 | .028 * |
|  |  |  | HIV+ User | 1.21 | .230 |
|  |  | HIV- User | HIV+ Nonuser | -1.49 | .140 |
|  |  |  | HIV+ User | 1.93 | .057 |
|  |  | HIV+ Nonuser | HIV+ User | 3.02 | .003 * |
|  | Right Insula | HIV- Nonuser | HIV- User | -.77 | .441 |
|  |  |  | HIV+ Nonuser | -2.31 | .024 * |
|  |  |  | HIV+ User | 1.64 | .105 |
|  |  | HIV- User | HIV+ Nonuser | -1.63 | .107 |
|  |  |  | HIV+ User | 2.27 | .026 * |
|  |  | HIV+ Nonuser | HIV+ User | 3.46 | < .001 * |
|  | Left Cerebellum | HIV- Nonuser | HIV- User | -1.10 | .275 |
|  |  |  | HIV+ Nonuser | -2.56 | .012 * |
|  |  |  | HIV+ User | 1.52 | .133 |
|  |  | HIV- User | HIV+ Nonuser | -1.61 | .112 |
|  |  |  | HIV+ User | 2.43 | .017 * |
|  |  | HIV+ Nonuser | HIV+ User | 3.58 | < .001 * |
|  | Right dlPFC | HIV- Nonuser | HIV- User | .12 | .905 |
|  |  |  | HIV+ Nonuser | -3.35 | .001 * |
|  |  |  | HIV+ User | 1.28 | .206 |
|  |  | HIV- User | HIV+ Nonuser | -3.37 | .001 * |
|  |  |  | HIV+ User | 1.14 | .259 |
|  |  | HIV+ Nonuser | HIV+ User | 4.09 | < .001 * |
| ***HIV Main Effect*** | Left Anterior Cingulate | HIV- | HIV+ | 3.73 | < .001 * |
|  | Right Cerebellum | HIV- | HIV+ | 3.24 | .002 * |
|  | Left Thalamus | HIV- | HIV+ | 3.10 | .003 * |
| ***Cannabis Main Effect*** | Right PFC | Nonuser | User | 3.94 | < .001 * |
|  | Right Superior Parietal Cortex | Nonuser | User | -3.49 | < .001 * |
|  | Right Cerebellum | Nonuser | User | 3.67 | < .001 * |
|  | Right Inferior Frontal Cortex | Nonuser | User | 3.45 | < .001 * |

*Note*. vPMC – Ventromedial premotor cortex; dlPFC – dorsolateral prefrontal cortex; PFC – prefrontal cortex; **p* < .05.

*Neural-Behavioral Correlations*

**Supplementary Table 3.** Neurobehavioral Relationships

|  | Reaction Time Flanker Effect (ms) | |
| --- | --- | --- |
| **Neural Measure** | *r* | *p* |
| Spontaneous γ Left M1 | .23 | .022 * |
| Spontaneous γ Right vPMC | .35 | < .001 * . |
| Spontaneous γ Right Anterior Insula | .28 | .004 * |
| Spontaneous γ Right dlPFC | .22 | .026 * |
| Spontaneous γ Left Cerebellum | .27 | .007 * |
| Oscillatory γ Left M1 | .11 | .286 |
| Oscillatory γ Right vPMC | -.07 | .504 |
| Oscillatory γ Right Anterior Insula | -.06 | .607 |
| Oscillatory γ Right dlPFC | .08 | .472 |
| Oscillatory γ Left Cerebellum | .11 | .300 |
| Spontaneous β Left M1 | .11 | .272 |
| Spontaneous β Right dPMC | .14 | .165 |
| Oscillatory β Left M1 | .01 | .921 |
| Oscillatory β Right dPMC | -.01 | .962 |

*Note*. M1 – Primary motor cortex; vPMC – ventral premotor cortex; dlPFC – dorsolateral prefrontal cortex; dPMC – dorsal premotor cortex; **p* < .05.


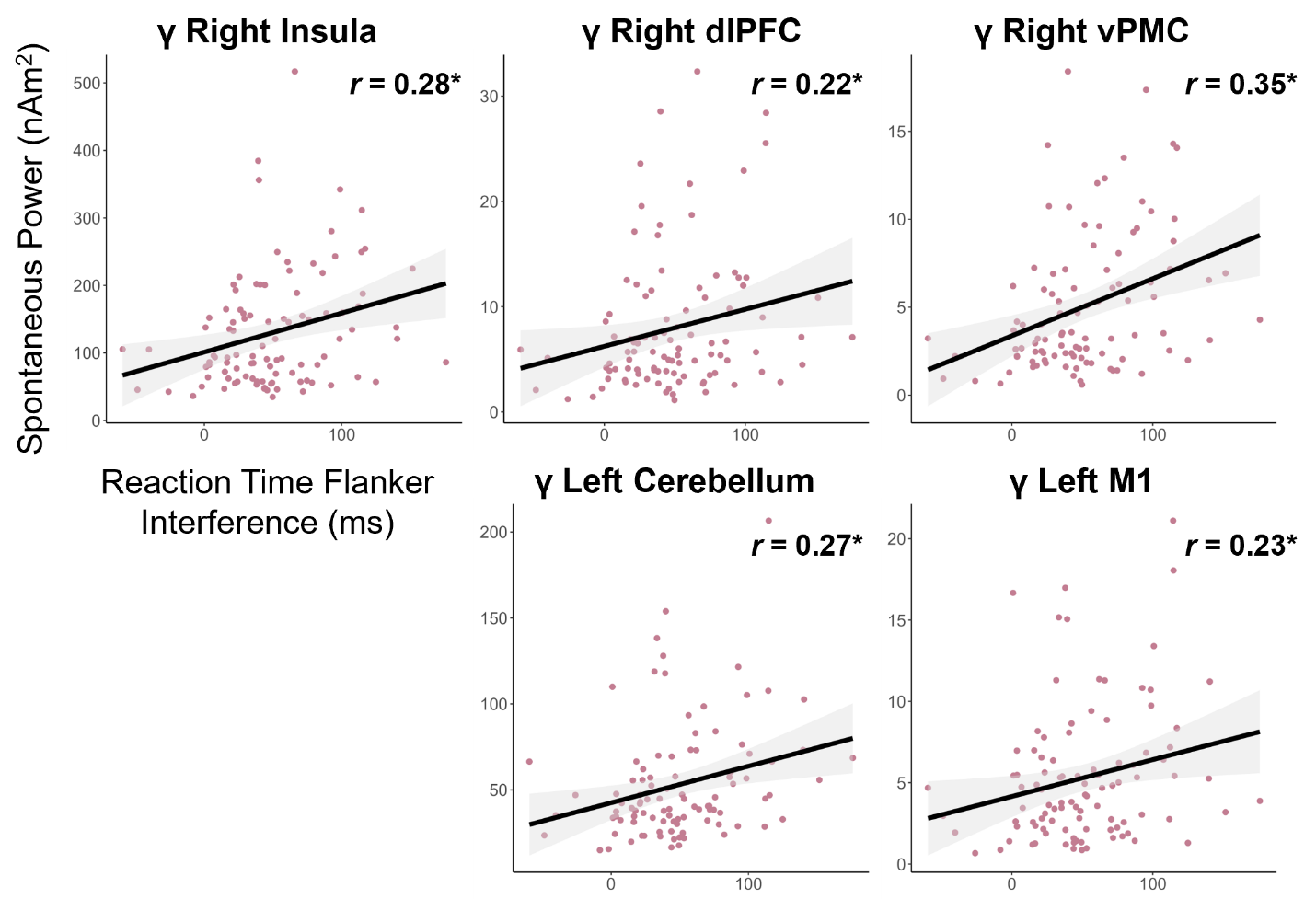


***Supplementary Figure 1. Spontaneous gamma activity scales with reaction time flanker interference***. Greater reaction time flanker interference (i.e., larger difference in reaction times between incongruent and congruent conditions) scaled with elevated spontaneous gamma (i.e., more abnormal) in the right insula (*r* = .28, *p* = .004), right dlPFC (*r* = .22, *p* = .026), right vPMC (*r* = .35, *p* < .001), left cerebellum (*r* = 27, *p* = .007), and the left M1 (*r* = 23, *p* = .022). **p* < .05.
